# Hepatocellular carcinoma associated with budd-chiari syndrome: imaging features and transcatheter arterial chemoembolization

**DOI:** 10.1186/1471-230X-13-105

**Published:** 2013-06-24

**Authors:** Feng-Yong Liu, Mao-Qiang Wang, Feng Duan, Qing-Sheng Fan, Peng Song, Yan Wang

**Affiliations:** 1 Department of Interventional Radiology, Chinese People’s Liberation Army General Hospital, 28 Fuxing Road, Beijing 100853, China

**Keywords:** Hepatocellular carcinoma, Budd–Chiari syndrome, Transcatheter arterial chemoembolization

## Abstract

**Background:**

Budd–Chiari syndrome (BCS) often leads to hepatocellular carcinoma (HCC). Transcatheter arterial chemoembolization (TACE) has been increasingly used to treat BCS patients with HCC. The purposes of this study were to illustrate imaging features in BCS patients with HCC, and to analyze the effects of TACE on BCS patients with HCC.

**Methods:**

246 consecutive patients with primary BCS were retrospectively studied. 14 BCS patients with HCC were included in this study. BCS were treated with angioplasty and/or stenting, and HCC were managed with TACE. Imaging features on ultrasonography, CT, MRI, and angiography and the serum AFP level were analyzed.

**Results:**

Inferior vena cava block and stricture of hepatic venous outflow tract more frequently occurred. Portal vein invasion was found in only 2 patients (14.2%). Imaging studies showed that most nodules of HCC were near the edge of liver, irregular, more than 3 cm in diameter, heterogeneous mass and solitary (≤3 nodules). HCC in patients associated with BCS was isointense or hypointense in nonenhanced CT images, and exhibited heterogeneous enhancement during the arterial phase and washout during the portal venous phase on enhanced CT and MRI. The serum AFP level significantly declined after TACE treatment.

**Conclusions:**

BCS patients with inferior vena cava block and stricture of hepatic venous outflow tract seems to be associated with HCC. A single, large, irregular nodule with a peripheral location appears to be HCC. TACE can effectively treat HCC in BCS patients.

## Background

The Budd–Chiari syndrome (BCS) is a heterogeneous group of disorders, which are characterized by hepatic venous outflow obstruction at the level of the hepatic venules, the large hepatic veins, the inferior vena cava (IVC), or the right atrium. Obstruction of hepatic venous outflow tract leads to sinusoidal congestion, ischemic injury to liver cells, and portal vein hypertension, subsequently leading to hepatic congestion with necrosis, regeneration, fibrosis, and liver cirrhosis [1-4]. Patients with BCS have been reported to be associated with hepatocellular carcinoma (HCC), the most common form of primary liver malignancy [5,6]. Several lines of evidence have shown that cirrhotic process following chronic hepatic congestion can result in the development of HCC in patients with BCS after excluding the influence of hepatitis viruses, alcoholism, autoimmune diseases or chemical intoxication [4,7-10].

BCS and HCC are often diagnosed on the basis of characteristic imaging findings on ultrasonography, CT, and MRI in combination with the clinical situation [6,11]. The serum a-fetoprotein (AFP) has also been used for diagnosis and screening of HCC [12]. There is an increasing acceptance of diagnosis of HCC using imaging techniques without biopsy [11,13,14]. However, the imaging features of HCC in patients with BCS have not been well established.

Several therapeutic options have been advocated to treat HCC, including transcatheter arterial chemoembolization (TACE), surgical resection, and radiofrequency ablation. TACE has been widely used in the treatment of HCC by interrupting the arterial supply to the tumor, thus leading to ischemic tumor necrosis [15]. Focused administration of chemotherapy during TACE increases the therapeutic effects and decreases systemic side effects of chemotherapy agents. TACE has been demonstrated to improve survival in patients with HCC, especially in patients with unresectable HCC [16,17]. It has been reported that TACE treatment results in an effective tumor response in about 60% of HCC patients associated with membranous obstruction of the inferior vena cava (MOVC) [9]. However, the effects of TACE treatment in HCC patients with primary BCS have not been well established yet.

In this study, we retrospectively investigated 14 consecutive HCC patients associated with BCS who underwent TACE treatment. The purpose of this study was to analyze the imaging features of HCC and changes in the serum AFP level after TACE treatment, to assess the risk factors associated with HCC, and to evaluate effectiveness of TACE treatment in these patients.

## Methods

### Patients

The study was approved by the Ethics Committee of Chinese People’s Liberation Army General Hospital, and all subjects gave their informed consent. We retrospectively studied 246 consecutive patients with a primary diagnosis of BCS, who were admitted to our hospital between March 1994 and May 2011. Date of diagnosis was the date of the first investigation when the criteria for diagnosis of BCS were fulfilled. Diagnosis of BCS followed the criteria by the European Group for the Study of Vascular Disorders of the Liver [6]. All patients underwent ultrasonography, magnetic resonance imaging (MRI), computed tomography (CT), and/or angiography, to examine the obstruction of hepatic veins, portal vein and/or IVC. The diagnosis of BCS was based on imaging showing an obstructed venous outflow tract. All patients had occlusion or stenosis of the hepatic veins and/or IVC. Patients with HBV and HCV infection, a history of alcoholism and chemical intoxication, and autoimmune diseases were excluded from this study. Fourteen BCS patients with HCC and fifty-one patients with benign nodules were included.

The diagnosis of HCC was followed the criteria by European Association for the Study of the Liver criteria for the diagnosis of hepatocellular carcinoma [11]. The diagnosis of HCC was based on imaging features on ultrasonography, CT and/or MRI and the serum AFP level [12,18]. Liver nodules were evaluated according to the following imaging features: shape, number, size, location, enhancement on arterial phase, washout on portal venous phase, tumor thrombus of the portal vein, and lung metastasis. All 246 patients underwent ultrasonography, 161 patients underwent enhanced MRI, and 85 patients underwent enhanced CT. Nodules were biopsied routinely if nodules had the at least one of the following features: 1) ≤ 3 in number; 2) ≥ 3cm in diameter, 3) heterogeneity; 4) washout on portal venous phase; 5) changes in on two successive imaging during the follow up period of at least 1 year; and 6) increase in the serum AFP levels. Benign nodules were considered when nodules had none of the above features, or malignancy was ruled out by histological examinations of the biopsied nodules. A nodule with two typical imaging features and a serum AFP level greater than 400 μg/L were the diagnosis criteria for HCC. Biopsies of liver nodules were performed to confirm the diagnosis of HCC. Table 1 summarizes the major clinical information of these 14 patients.

**Table 1:** Clinical features of 14 HCC patients associated BCS

**Clinical features**	**Data**
Male: female	8:6
Mean age (range)	44.69 years (27–60 years)
Duration of symptoms (range)	14.8 years (0.6-28 years)
Hepatic vein block (n)	0
Inferior vena cava block (n)	8
Both Hepatic vein and Inferior vena cava block (n)	6
Stricture: Obstruction	10:4

### Imaging

Computer tomography (CT) was performed by a 16-slice spiral CT scanner (GE LightSpeed). Nonionic radiographic contrast medium Ultravist or Omnipaque (90–100 ml) was administered intravenously at the rate of 3.5 ml/sec. CT images were obtained during hepatic arterial phase (25 sec after the injection of contrast medium) and the portal venous phase (60 sec after the injection of contrast medium). The magnetic resonance imaging (MRI) was produced by a 1.5-T MRI device (GE signa). MRI protocol included T1-weighted imaging (T1WI), T2-weighted imaging (T2WI), and gadolinium-enhanced MRI. After bolus injection of gadolinium-DTPA at a rate of 2.5 ml/sec, gadolinium-enhanced MR angiography were performed during arterial phase (14–16 sec after the injection of contrast medium), the venous phase (10–15 sec after arterial phase), and the parenchymal phase (120–150 sec after venous phase).

Untrasonograms were obtained with a 1024 × 1024 matrix at a frame rate of 3–6 sec by using a digital subtraction angiographic (DSA) unit (GE LCV Plus) and a digital flat-panel system (INNOVA 3100/4100). Both DSA and mask images were recorded. For celiac artery and superior mesenteric angiography, a 4F catheter was placed at the celiac artery and superior mesenteric artery, and nonionic radiographic contrast medium Ultravist (25 ml) was administered at the rate of 5 ml/sec. For inferior vena cava angiography, a 4F inferior vena cava catheter was placed at the hepatic segment of inferior vena cava, and nonionic radiographic contrast medium Ultravist (30 ml) was administered at the rate of 15 ml/sec. For hepatic venous angiography, a Cobra, or Yashiro catheter was placed at the hepatic vein, and nonionic radiographic contrast medium Ultravist (9 ml) was administered at the rate of 3 ml/sec.

### Interventional treatment

Transcatheter arterial chemoembolization (TACE) were performed after visceral vascular evaluation on the hepatic arterial anatomy, the arterial supply of the tumor, and any ateriovenous shunting. Selective catheterization of celiac artery-hepatic artery was performed by a 4F catheter, and fluorouracil (500–750 mg) was administered. Selective catheterization of branches of hepatic artery was achieved by a 3F microcatheter, and a mixed solution containing epirubicin (20–50 mg), oxaliplatin (100–150 mg), mitomycin (8-12 mg) and lipiodol (5–20 ml) was administered.

Occlusion or stenosis of inferior vena cava and hepatic vein was treated with balloon catheter dilation. The balloon catheter was inserted into inferior vena cava and hepatic vein through femoral vein and/or jugular vein. A balloon catheter of 20–25 mm in diameter and 40 mm in length was used for inferior vena cava, and a balloon catheter of 8–12 mm in diameter and 40 mm in length was used for hepatic vein.

### Follow-up

All patients underwent ultrasonography, CT and/or MRI during the follow-up. Serum AFP was also tested during the follow-up. The imaging features of HCC and the effects of treatment were evaluated.

### Statistical analysis

Analyses were performed using SPSS 17.0. Quantitative data were expressed as mean and range. Student *t* test was used to compare the difference between before and after treatment of quantitative data. Categorical data were compared using the chi-squared test or Fisher’s exact test. Probability values less than 0.05 were considered statistically significant.

## Results

### Clinical features

The clinical features of 14 HCC patients associated with BCS are shown in Table 1. Eight (57.14%) of fourteen patients were male, and six (42.86%) patients were female. The mean age was 44.69 years (range, 27–60 years). HCC was diagnosed with imaging features (ultrasonography, CT and/or MRI) and serum AFP level in 10 patients, and with histological examinations in 4 patients after surgical removal of the nodule (biopsy for 2 patients, surgical resection for 1 patient, and liver transplantation for 1 patient). The histological findings of the four samples showed hepatocellular carcinoma and cirrhosis in adjacent parenchyma. The mean duration between diagnosis of BCS and HCC was 14.8 years (range, 0.6-28 years). Inferior vena cava block alone was found in 57.14% (8/14) of patients, and hepatic vein and inferior vena cava block was observed in 42.86% (6/14) of patients. Venous outflow tract stricture and obstruction was found in 71.43% (10/14) and 28.57% (4/14) of patients, respectively.

The serum AFP level was normal in 2 patients with HCC (1.86 μg/L and 15.09 μg/ L), whereas the mean level was 5624.58 μg/L (range, 63.9–20000 μg/L) in the other 12 patients. The tumor size was 5 cm and 3 cm for the 2 patients with normal AFP level, whereas the mean tumor size was 9.6 cm (range, 1.2-20 cm) in the other 12 patients. In addition, benign nodules were found in 51 patients with BCS. The serum AFP levels were stable and normal (< 20 μg/L) in all these patients.

Portal vein invasion occurred in 2 of 14 HCC patients associated with BCS. In contrast, portal vein invasion was found in 129 of 200 HCC patients caused by hepatitis viruses. The frequency of occurrence of portal vein invasion was significantly different between the two groups of HCC patients (χ^2^ = 11.8611, *P* = 0.0006).

### Imaging features of hepatocellular carcinoma

**Figure 1:**
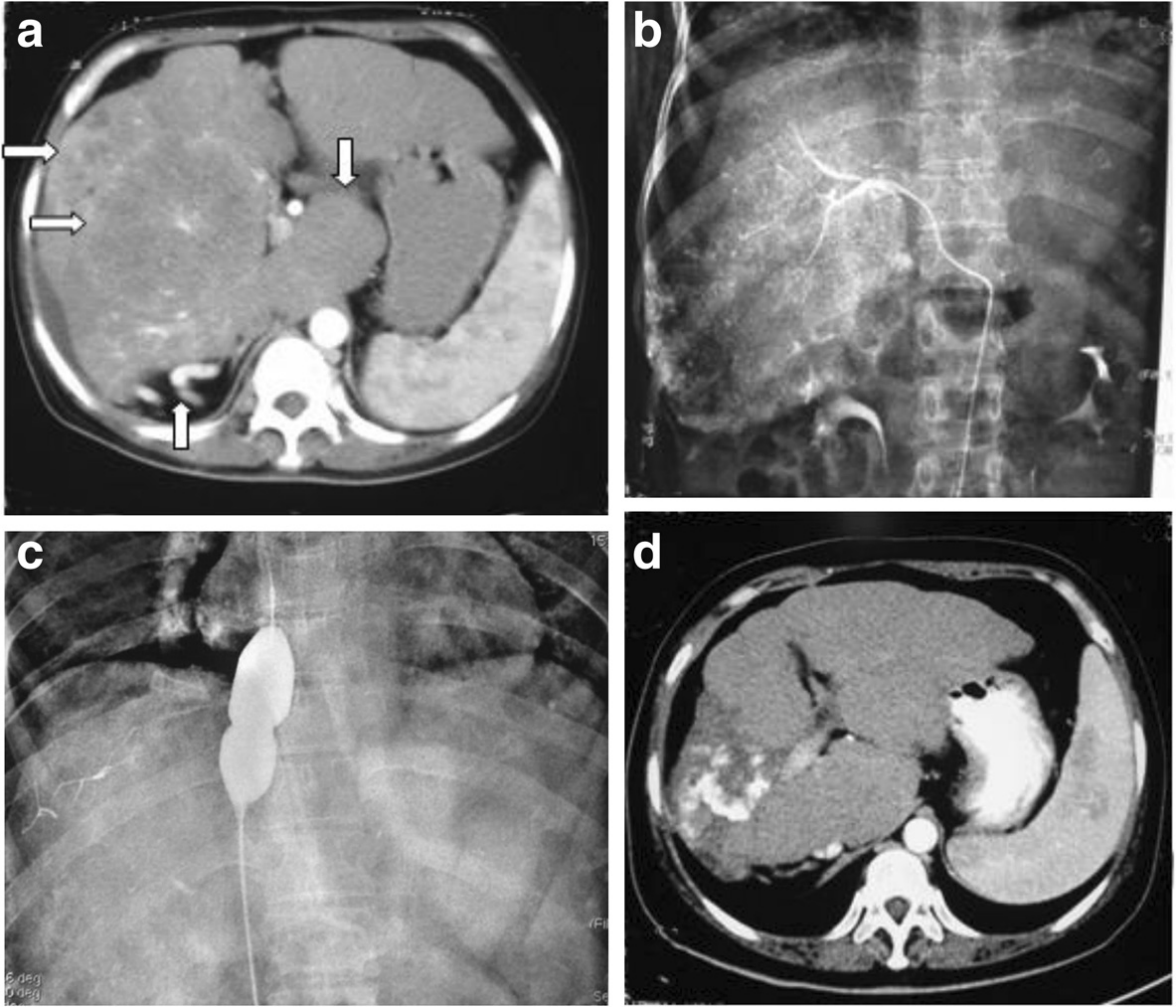
**45-year-old woman with HCC in BCS.** (**a**) Contrast-enhanced transverse CT scan showing two large heterogeneous enhanced nodules (⇨) in the right liver during arterial phase and enlarged caudate lobe (⇩), ventral varicosities (⇧), liver cirrhosis and mild ascites. (**b**) The two nodules were given TACE. (**c**) The blocked inferior vena cava was given angioplasty. (**d**) CT scan showing that the nodules were obviously shrinked and obtained stable effect after 4 TACE and 1 angioplasty during the 22-month treatment. Lipiodol was not located equably in the nodules.

**Figure 2:**
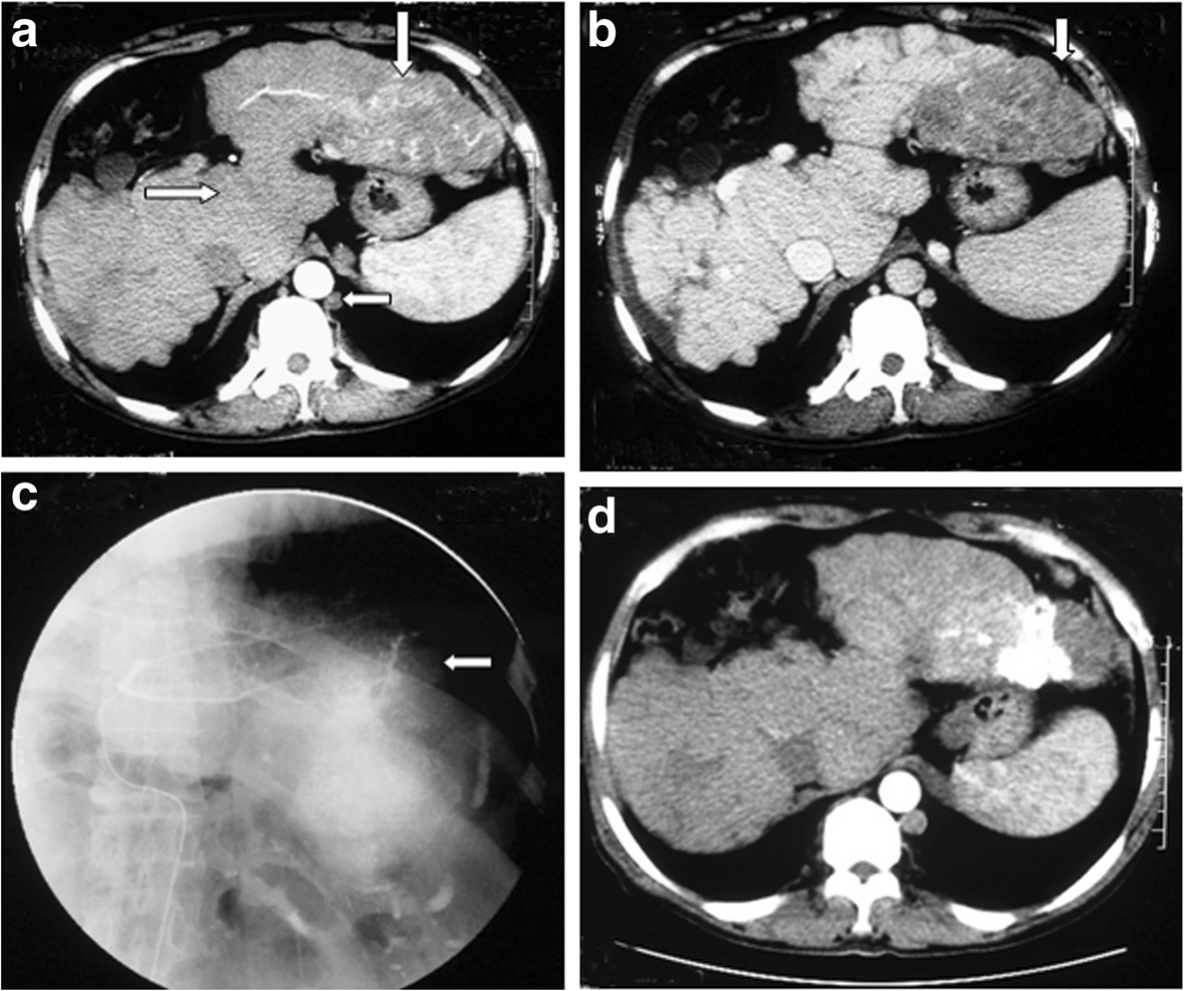
**50-year-old man with HCC in BCS.** (**a**) Contrast-enhanced transverse CT scan showing a large heterogeneous enhanced nodule (⇩) in the left liver during arterial phase, and enlarged caudate lobe (⇨), cirsoid semi-azygous vein (⇦), and liver cirrhosis. (**b**) Washout of the nodule on portal venous phase (⇩). (**c**) Angiography showing heterogeneous, contorted and enlarged tumorous vein and tumor staining in the left liver (⇦) during arterial phase. TACE was given. (**d**) CT scan showing that the nodule was obviously shrinked and obtained stable effect 2 months after TACE treatment. Lipiodol was not located equably in the nodule.

**Figure 3:**
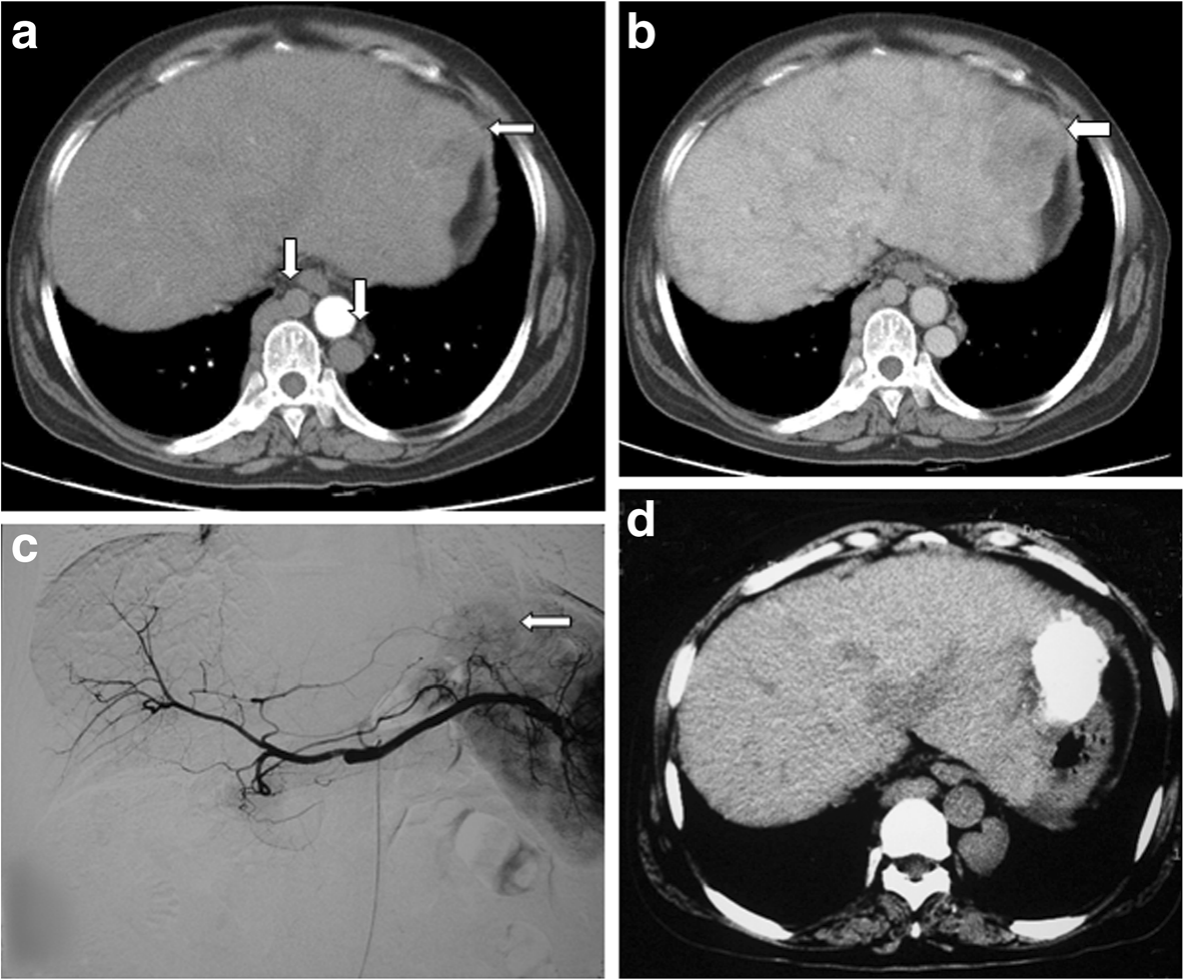
**51-year-old woman with HCC in BCS.** (**a**) Contrast-enhanced transverse CT scan showing a large mild heterogeneous enhanced nodule(⇦) in the left liver during arterial phase and obvious cirsoid azygous vein and semi-azygous vein (⇩) and liver congestive cirrhosis. (**b**) Washout of the nodule on portal venous phase (⇦). (**c**) Angiography showing heterogeneous and contorted tumorous veins in the left liver (⇦) during arterial phase. TACE was given at the first time. (**d**) Contrast-enhanced transverse CT scan showing that the nodule was obtained stable effect after 2 TACE treatments for 14 months. Lipiodol was located equably in the nodule.

Diagnosis of BCS was based on ultrasonography, CT and/or MRI (Figure 1, Figure 2A,B, Figure 3A,B). The final diagnoses were based on angiography. HCC was diagnosed with CT in 10 patients and with CT and MRI in 4 patients, and was confirmed with DSA before TACE in all 14 patients. Lung metastasis was found in 3 patients. The imaging features of 14 patients are summarized in Table 2. All patients with HCC had three or fewer nodules, and 10 patients had only one nodule. The largest nodule, usually located near the edge of the liver, was selected to evaluate the size, location, and shape of the nodule, if the patient had more than one nodule. Mean size of the nodule was 8.75 cm (range, 1.2–20 cm) in diameter.

**Table 2:** Imaging features of 14 HCC patients associated with BCS

**Imaging features**	**Data**
Liver cirrhosis (n)	14
Number of nodules	21
Mean size (range)	8.75 cm (1.2-20 cm)
Location (left lobe:right lobe)	8:6
Shape (irregular:regular)	11:3
Invasion of the portal vein	2
Lung metastasis	3
Nonenhanced phase	Isointense or hypointense
Enhancement	
Arterial phase	Heterogeneous enhancement
Portal venous phase	washout

The largest nodule was selected to evaluate the size, location and shape of the nodule, if the patient had more than one nodule.

Nonenhanced CT images showed that all 21 nodules in the 14 patients with HCC were isointense or hypointense relative to the surrounding parenchyma. The 20 nodules exhibited irregular and heterogeneous enhancement on the arterial phase. Only one nodule with tumor size of 1.2 cm (serum AFP level of 84.6 μg/L) showed a regular enhancement on the arterial phase. Washout on portal venous phase occurred in all nodules, and a central scar was not found in any nodules. Portal vein invasion was found in 2 of 14 patients.

Axial T1-weighted MR images obtained during the arterial phase showed that all nodules exhibited an irregular and heterogeneous enhancement. Axial T1-weighted MR images obtained during the portal venous phase showed that washout occurred in all nodules. On T2-weighted MR images, these nodules were hyperintense or isointense.

In contrast, benign nodules were regular, homogeneous, numerous, and small, compared with nodules in HCC. Only two benign nodules had a diameter of >3 cm. The benign nodules showed homogeneous enhancement on the arterial phase and slight hyperintensity on portal venous phase.

Cirrhosis was found in the adjacent parenchyma in all patients with HCC, and in 56.9% (29/51) of patients with benign nodules. There was a significant difference in the occurrence of cirrhosis between patients with HCC and patients with benign nodules (χ^2^ = 7.3041, *P* = 0.0069). In addition, 43.1% (22/51) of patients with benign nodules had hepatic congestion and hepatomegaly.

### Treatment

The treatment of 14 patients with HCC is shown in Table 3. Nine patients underwent angioplasty for hepatic vein and/or inferior vena cava block. Stenting was performed in one patient to treat inferior vena cava block (Figure 1B,C). There were 25 TACE treatments for the 14 patients (range, 1–4 times per patient) (Figure 1B,C, Figure 2C, Figure 3C). One patient, who underwent angioplasty and stenting 8 years ago, was treated with TACE and angioplasty when HCC and recurrent inferior vena cava stricture were found. Five patients underwent angioplasty when they were treated with TACE at the first time. Four patients underwent angioplasty when they were treated with TACE at the second time. Four patients were treated with only TACE. After TACE treatment, three patients underwent radiofrequency treatment, and two patients underwent surgical resection of the tumor. One patient underwent liver transplantation at 3 months after surgical removal of the tumor.

**Table 3:** Treatment of HCC and/or BCS in 14 patients with HCC

**Treatment**	**HCC**	**BCS**
Angioplasty		9
Stenting		1
TACE	25	
Surgical resection	2	
Liver transplantation	1	
Radiofrequency	3	

### Fellow-up

All patients with HCC were followed up with an average period of 33.46 months (range, 2–71 months) after TACE. Lipiodol distributed in the HCC evenly in 12 of 14 patients (Figure 1D, Figure 2D, Figure 3D). One patient showed progression of HCC during the follow-up period of 25 months after TACE. The other one lost to follow-up after 2 months. The serum AFP level significantly declined from 4760.57 μg/L before TACE treatment to 60.13 μg/L during the follow-up period of >1 year after TACE treatment (*t* = 2.336, *P* = 0.0377).

Other patients without HCC were followed up for 1-15 years (median, 7.8 years), and no HCC were found.

## Discussion

HCC has been regarded as one of the major complications of BCS, and appears to develop chronically with the progression of BCS [10,19]. The prevalence of HCC in patients with BCS varies in different regions. The incidence is high in Japan (41%), South Africa (48%), and the United States (25%) [20-22]. The incidence in China has not been reported. In this study we found that the cumulative incidence of HCC was 5.69%, which was lower than that reported in Japan, South Africa and the United States. The incidence of HCC in patients with BCS is similar to that in patients with other chronic liver diseases (4–17%) [23].

The accurate pathogenesis of HCC in patients with BCS has not been elucidated. It is believed that hepatic congestion caused by obstruction of hepatic venous outflow tract can lead to hepatic necrosis, fibrosis and cirrhosis, which contribute to the pathogenesis of HCC in patients with BCS [24]. This idea is supported by our findings of cirrhotic change in liver parenchyma adjacent to HCC in all patients with HCC. In addition, the occurrence of cirrhosis was significantly higher in patients with HCC than that in patients with benign nodules, further suggesting that cirrhosis may contribute to the development of HCC.

It has been reported that patients with long-standing inferior vena cava block have a 70-fold higher risk of developing HCC than those with pure hepatic vein block [3]. In agreement with this report, we found that patients with inferior vena cava block were more frequently complicated with HCC than those with single hepatic vein block. Patients with single hepatic vein block were not associated with HCC in our study. In addition, stricture of hepatic venous outflow tract (in 10 patients) was more frequently associated with HCC than obstruction of hepatic venous outflow tract (in 4 patients). We also found that the frequency of occurrence of portal vein invasion was significantly lower in HCC patients with BCS than that in HCC patients with hepatitis, suggesting that extrahepatic metastases is less likely to occur in HCC patients with BCS.

Imaging studies in combination with clinical information are often essential for a definitive diagnosis of HCC, and can help to distinguish it from other hypervascular masses [25]. In this study, we found imaging features of HCC in patients with BCS, which are useful to distinguish HCC from benign hypervascular regenerative nodules. HCC in patients associated with BCS was isointense or hypointense in nonenhanced CT images, and exhibited heterogeneous enhancement on arterial phase and washout on portal venous phase. Axial T1-weighted images showed that all nodules exhibited irregular and heterogeneous enhancement during the arterial phase, and washout during the portal venous phase. T2-weighted images showed that these nodules were hyperintense or isointense. These imaging features are consistent with previous reports in patients with HCC [25-27]. In addition, the location, shape, size, number of nodules have a high values to distinguish HCC from benign nodules [3,25]. Most nodules of HCC were usually near the edge of liver, irregular, more than 3 cm in diameter, heterogeneous mass and solitary (≤3 nodules). Different and almost opposite features were found in benign nodules.

The serum AFP level can be used for diagnosis and screening of HCC [12]. An increase in AFP level (>20 μg/L) was found in 12 of 14 patients with HCC, but not in any patients with benign nodules, suggesting that the serum AFP level is valuable for the diagnosis of HCC. In addition, the serum AFP level significantly declined after TACE treatment, suggesting that the serum AFP level can be used to evaluate the efficacy of TACE treatment.

Shin et al. suggested that HCC patients associated BCS were susceptible to aggressive intervention such as TACE [4]. In agreement with this report, we found that the serum AFP level significantly declined during the follow-up. In addition, angioplasty and stenting were performed to treat hepatic vein and/or inferior vena cava block in these patients. Elimination of hepatic vein and/or inferior vena cava block can result in an improvement of liver a reduction by reducing the hepatic sinusoidal pressure, thus preventing the occurrence of HCC [1]. However, since angioplasty and stenting are only performed in nine patients in different ways, it remains unclear whether angioplasty and stenting can reduce the occurrence of HCC, and whether angioplasty and stenting can improve the effect of TACE. Until now, there is no consensus about the methods and timing of the treatment for HCC patients with BCS. Large cohort studies are needed to confirm the values of these therapeutic methods in the future.

The prognosis in patients with HCC remains poor because the high rate of tumor recurrence or the development of new tumor [28,29]. TACE has been shown to improve survival of patients with HCC in a randomized control trial [16]. It has been reported that the 3- and 5-year survival rates of patients receiving TACE was 26-47% [16,17,30,31] and 16-26% [32-34], respectively. Because of the low incidence of BCS patients with HCC, no studies with a large patient population have been investigated the long-term survival of BCS patients with HCC receiving TACE. In a study of 20 HCC patients with membranous obstruction of the inferior vena cava who received TACE , the 3- and 5 year survival rate was 61% and 46%, respectively [9]. In the present study, all patients survived during the mean follow-up period of 33.46 months (range, 2–71 months). However, since some patients were not followed up for more than one year, the survival rate was not determined in our study.

## Conclusions

In this study, we investigated the imaging features of HCC and changes in the serum AFP level after TACE treatment HCC patients associated with BCS. A single, large, irregular nodule with a peripheral location appears to be HCC. Inferior vena cava block and stricture of hepatic venous outflow tract more frequently occur in BCS patients with HCC. TACE is an effective treatment of HCC in patients with BCS.

## Abbreviations

BCS: Budd–Chiari syndrome; IVC: Inferior vena cava; HCC: Hepatocellular carcinoma; AFP: A-fetoprotein; TACE: Transcatheter arterial chemoembolization; MOVC: Membranous obstruction of the inferior vena cava; MRI: Magnetic resonance imaging; CT: Computed tomography; DSA: Digital subtraction angiographic.

## Competing interests

The authors have no relevant conflicts of interest to disclose.

## Authors’ contributions

FL and MW designed and performed the research, analyzed data, and wrote the paper. FD, QF, PS, and YW performed the research, analyzed data, and edited the manuscript. All authors read and approved the final manuscript.

## Pre-publication history

The pre-publication history for this paper can be accessed here:

http://www.biomedcentral.com/1471-230X/13/105/prepub
